# Vaginal rings with exposed cores for sustained delivery of the HIV CCR5 inhibitor 5P12-RANTES

**DOI:** 10.1016/j.jconrel.2019.02.003

**Published:** 2019-03-28

**Authors:** John W. McBride, Peter Boyd, Nicola Dias, David Cameron, Robin E. Offord, Oliver Hartley, Vicky L. Kett, R. Karl Malcolm

**Affiliations:** a School of Pharmacy, Queen's University Belfast, Belfast BT9 7BL, UK; b Envigo, Huntingdon, Cambridgeshire, UK; c Mintaka Foundation for Medical Research, Geneva, Switzerland; d Department of Pathology and Immunology, Faculty of Medicine, University of Geneva, Geneva, Switzerland

**Keywords:** HIV microbicide, Sustained release, Silicone elastomer, Sheep pharmacokinetics, Protein drug delivery

## Abstract

Antiretroviral-releasing vaginal rings are at the forefront of ongoing efforts to develop microbicide-based strategies for prevention of heterosexual transmission of the human immunodeficiency virus (HIV). However, traditional ring designs are generally only useful for vaginal administration of relatively potent, lipophilic, and small molecular weight drug molecules that have sufficient permeability in the non-biodegradable silicone elastomer or thermoplastic polymers. Here, we report a novel, easy-to-manufacture ‘exposed-core’ vaginal ring that provides sustained release of the protein microbicide candidate 5P12-RANTES, an experimental chemokine analogue that potently blocks the HIV CCR5 coreceptor. *In vitro* release, mechanical, and stability testing demonstrated the utility and practicality of this novel ring design. In a sheep pharmacokinetic model, a ring containing two ¼-length excipient-modified silicone elastomer cores – each containing lyophilised 5P12-RANTES and exposed to the external environment by two large windows – provided sustained concentrations of 5P12-RANTES in vaginal fluid and vaginal tissue between 10 and 10,000 ng/g over 28days, at least 50 and up to 50,000 times the reported *in vitro* IC50 value.

## Graphical abstract

**Figure:**
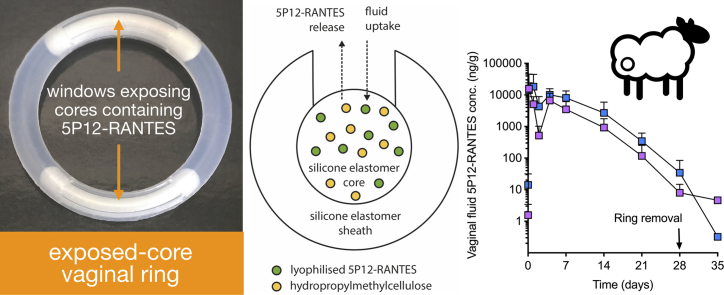

## 1 Introduction

**Fig. 1:**
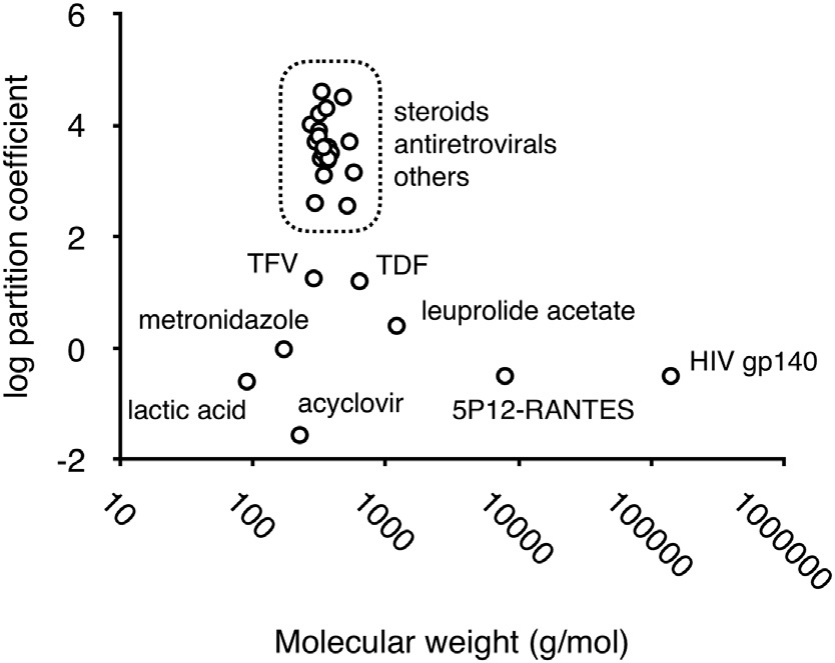
Plot of log partition coefficient (logP; experimental or calculated values) *vs.* molecular weight for drug molecules in marketed vaginal rings or having previously been considered for formulation in vaginal rings. Plot symbols inside the dashed box include estradiol, ethinyl estradiol, etonogestrel, estradiol-3-acetate, dapivirine, progesterone, levonorgestrel, maraviroc, MIV-150, oxybutynin, segesterone acetate (Nestorone®), norethindrone acetate, ulipristal acetate, medroxyprogesterone acetate, UC781, danazol, MC1220, CMPD-167, drosperinone, nomegestol acetate, and vicriviroc. Molecules outside the dashed box (see labels within the figure) are those currently being considered for vaginal ring formulations and that, due to physiochemical constraints, generally require novel formulation approaches.

Vaginal rings for controlled release of drug substances to the human vagina were first described in 1970 [[1], [2], [3]] following earlier reports demonstrating drug permeation through polysiloxane tubes when implanted in the ventricular myocardium of dogs [4,5], subdermally in ewes [6] and subdermally in rats [7]. Seven vaginal ring products have since reached market, five of which are fabricated from silicone elastomers (Estring®, Femring®, Progering®, Fertiring® and Annovera®) and two from thermoplastic polymers (Nuvaring® and Ornibel®) (Table 1). Also, an antiretroviral-releasing silicone elastomer vaginal ring for HIV prevention is presently under review by the European Medicines Agency [8,9], while various other drug-releasing ring devices are undergoing clinical testing (Table 1). The active pharmaceutical ingredients in these vaginal ring products are all highly potent, small molecular weight (< 540 g/mol), lipophilic (log *P* > 2) steroids or antiviral molecules (Table 1, Fig. 1) that can readily permeate the hydrophobic silicone elastomers and thermoplastic polymers to offer clinically significant release rates. In general, for molecules having physicochemical characteristics outside the limits defined by the dashed box in Fig. 1, conventional ring designs and/or polymeric materials are usually not practical due to significant constraints in drug permeability [10,11].

**Table 1:** Summary characteristics of marketed vaginal rings and rings in clinical development.

Product name	vaginal ring type; polymer^a^	Drug(s)	Clinical indication; duration of action	Developer	Stage
Estring®	reservoir; silicone	17β-estradiol	estrogen replacement therapy; 90 days	Pfizer	Marketed
Nuvaring®	reservoir; EVA	etonogestrel and ethinyl estradiol	combination contraception; 21 days	Merck Sharp & Dohme	Marketed
Femring®	reservoir; silicone	17β-estradiol-3-acetate	estrogen replacement therapy; 3 months	Warner Chilcott	Marketed
Progering®	matrix; silicone	progesterone	postpartum contraception; 1 year	Population Council	Marketed
Fertiring®	matrix; silicone	progesterone	hormonal supplementation and pregnancy maintenance; 3 months	Population Council	Marketed
Ornibel®	reservoir; EVA and TPU	etonogestrel and ethinyl estradiol	combination contraception; 21 days	Exeltis Healthcare	Marketed
Annovera®	reservoir; silicone	Nestorone® and ethinyl estradiol	combination contraception; one year	Population Council	Approved
–	matrix; silicone	dapivirine	HIV prevention; 30 days	IPM	Undergoing review
–	matrix; silicone	dapivirine and maraviroc	HIV prevention; 30 days	IPM	Phase I
–	matrix; silicone	dapivirine and levonorgestrel	HIV prevention and contraception; 90 days	IPM	Phase I
–	reservoir; TPU	tenofovir	HIV and HSV-2 prevention; 90 days	CONRAD	Phase I
–	reservoir; EVA	vicroviroc and MK-2048	HIV prevention; 28 days	Merck Sharp & Dohme	Phase I
–	reservoir; silicone	ulipristal acetate	contraception; 3 months	Population Council	Phase 1/2

a EVA – ethylene vinyl acetate copolymer; TPU – thermoplastic polyurethane.

There is considerable interest in developing new vaginal ring designs for sustained or controlled release of large molecular weight and/or hydrophilic drug molecules, and especially therapeutic peptides, proteins, antibodies, and nucleic acids. Much of the innovation in vaginal ring design during recent years has been driven by efforts to develop microbicidal vaginal rings for prevention of sexual transmission of the human immunodeficiency virus (HIV) and multipurpose prevention technology (MPT) rings for simultaneous prevention of HIV, pregnancy, and/or sexually transmitted infections [[11], [12], [13]]. By using more innovative vaginal ring designs and alternative polymer materials, researchers have demonstrated effective release of relatively hydrophilic small-molecule antiviral molecules (*e.g.* tenofovir [[14], [15], [16], [17]], tenofovir disoproxil fumarate [[18], [19], [20], [21]], acyclovir [17,[22], [23], [24]], emtricitabine [19,20]), peptides (*e.g.* leuprolide acetate [25], T-1249 [26]), proteins (*e.g.* HIV glycoprotein gp140 [27], antibodies [[28], [29], [30]]), hydrophilic polymers (*e.g.* carrageenan [31,32]) and metal-containing actives (*e.g.* cisplatin [33]; zinc acetate [31,32]).

However, many of these newer ring designs require complex multi-step manufacturing processes that will likely prove difficult and expensive to scale. For example, the leuprolide-releasing ethylene vinyl acetate (EVA) ring described by Kimball et al. comprises ten distinct manufacturing steps: (i) washing of EVA beads to remove monomeric vinyl acetate; (ii) vacuum drying of beads; (iii) cryogenic milling of EVA granules to powder; (iv) preparation of ethanolic solution of EVA and leuprolide; (v) solvent casting of the ethanolic mixture; (vi) solvent evaporation; (vii) milling of the EVA/drug mixtures to form a powder; (viii) extrusion of the leuprolide acetate/EVA granulation mixture; (ix) pelletization of the mixtures; and finally (x) manufacture of the rings by injection molding [25]. The widely reported pod-insert vaginal rings [17,19,21,23,30,[34], [35], [36]] – comprising multiple individual polymer-coated drug cores embedded in a ring body, each core connected to the external environment through a preformed delivery channel – are similarly complex and time-consuming to manufacture, involving at least nine distinct steps: (i) compaction of drug powder to form a core; (ii) drop-coating of cores using a polylactic acid solution; (iii) fabrication of silicone elastomer ring bodies *via* injection molding; (iv) creation of delivery channels by mechanical punching; (v) trimming of sprue material and flashing; (vi) cleaning of silicone with ethanol to remove debris from the molding process; (vii) manual placement of a drug-loaded pod into each cavity; (viii) backfilling of the pod cavity with non-medicated silicone elastomer; (ix) filling of any unused pod cavities [17]. There is, therefore, a need for new vaginal ring designs for sustained/controlled release of hydrophilic or macromolecular drugs that can be easily manufactured using more conventional and scalable processes.

5P12-RANTES ( MW 7904.8 g/mol) – a fully recombinant chemokine analogue that potently blocks the HIV CCR5 coreceptor – is being developed as both a vaginal and rectal microbicide for prevention of sexual transmission of HIV [[37], [38], [39]]. Previous studies have reported complete protection against vaginal challenge with simian/human immunodeficiency virus (SHIV) in rhesus macaques [40] and encouraging pharmacokinetic data in a sheep model following administration of vaginal gels containing 5P12-RANTES [41]. Unusually for a protein therapeutic, the high thermal and biological stability [37,39,41], coupled with a scalable low-cost cGMP process for production of clinical grade material [42], make 5P12-RANTES a viable candidate for formulation in a vaginal ring device.

Here, for the first time, we report a new ‘exposed-core’ vaginal ring design and demonstrate its potential for sustained release of proteins, first using lysozyme as a model protein, and then using 5P12-RANTES as a potent experimental HIV microbicide. Manufactured from silicone elastomer and common water-swellable pharmaceutical excipients using simple, scalable and well-established injection molding technologies, the ring offers certain advantages over previously reported ring designs for vaginal administration of hydrophilic and macromolecular drugs.

## 2 Materials and methods

### 2.1 Materials

5P12-RANTES solution (cGMP grade; 6.4 mg/mL in 1.7 mM acetic acid; pH 4.0) was supplied to Queen's University Belfast by the Mintaka Foundation for Medical Research, Geneva Switzerland [42]. 10 and 120 kDa molecular weight hydroxypropylmethylcellulose (HPMC), lysozyme, acetonitrile and trifluoroacetic acid (TFA) were purchased from Sigma-Aldrich (UK). DDU-4320 silicone liquid rubber was obtained from Nusil Technology (Carpinteria, CA, USA) and the pre-stained molecular weight marker from Life Technologies (Thermo Fisher Scientific, UK). 1× tris/glycine/SDS buffer, Laemmli sample buffer and 20% Mini-PROTEAN TGX precast gels were purchased from Bio-Rad, UK.

#### 2.1.1 Sieving of HPMC material

HPMC powder (molecular weight: 120 kDa) was separated into four particle size fractions using stacked analytical sieves having 125 μm, 90 μm and 53 μm mesh sizes. HPMC that remained in the 125 μm sieve was classified as the >125 μm fraction, powder that remained in the 90 μm sieve as the 90–125 μm fraction, material in the 53 μm sieve was designated as 53–90 μm and the material that passed through all sieves was the <53 μm fraction.

#### 2.1.2 Freeze-drying of 5P12-RANTES

5P12-RANTES solution was freeze-dried to produce a lyophilised powder. 5P12-RANTES was aliquoted into flat bottomed glass petri dishes (diameter 200 mm) and freeze-dried (AdVantage Pro BenchTop Freeze Dryer, VirTis, Gardiner, NY, USA) using the following parameters: 5 to −40 °C ramp for 1 h followed by an additional freeze for 2 h. The condenser was then set to −50 °C and a chamber pressure of 50 mTorr. Shelf temperature was gradually raised to 20 °C over 27 h, with a coinciding increase in chamber pressure from 120 to 190 mTorr. Further details are provided in the supplementary data (Table S1).

#### 2.1.3 Vaginal ring manufacture

**Fig. 2:**
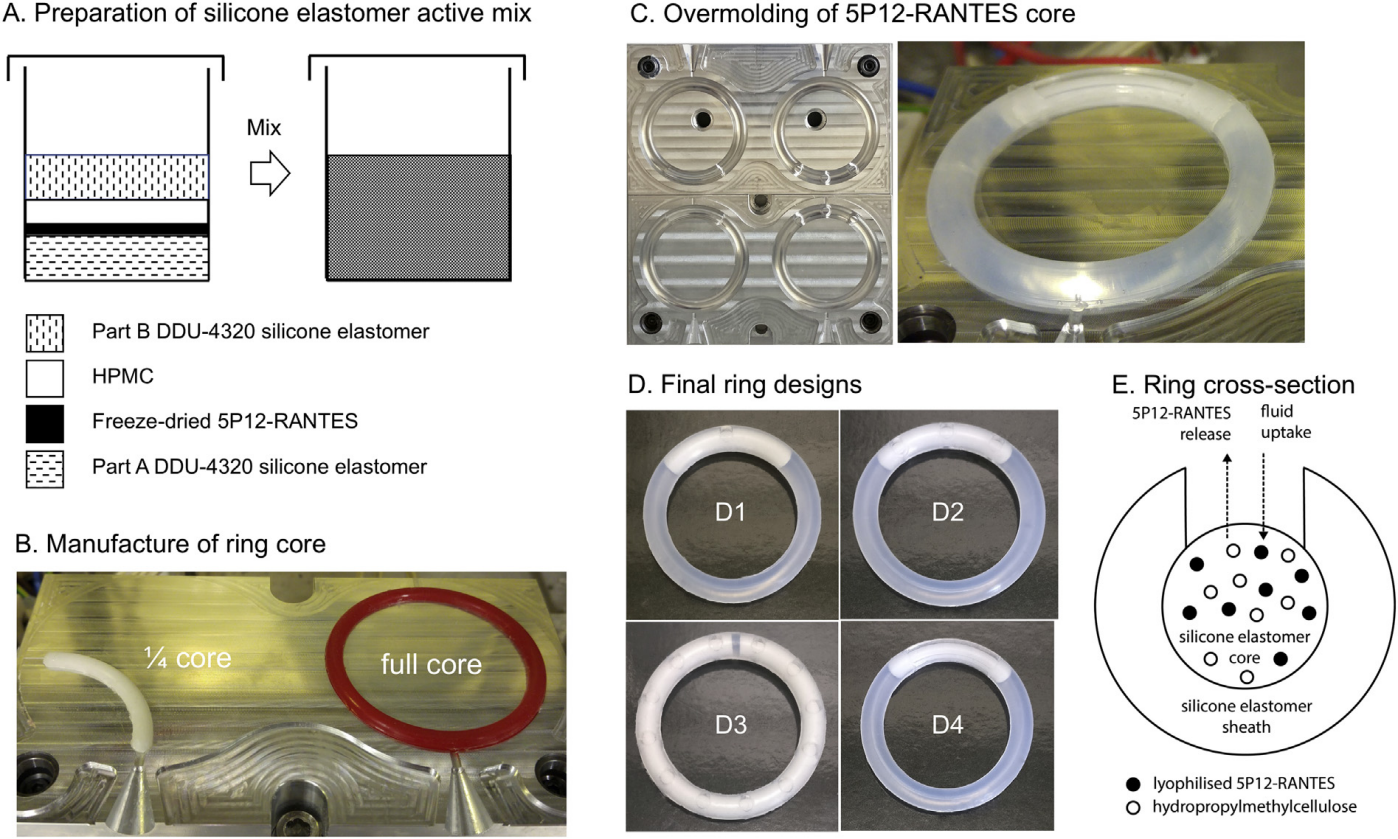
Description of the steps required for manufacture of an exposed-core vaginal ring. A – Preparation of silicone elastomer active mix comprising Parts A and B of the DDU-4320 silicone elastomer, the freeze-dried 5P12-RANTES and hydroxypropyl methylcellulose (HPMC). B – Mold tool for production of ¼- or full-length 5P12-RANTES cores. C – Cores are placed into custom mold tools having raised pins to position and support the core. Subsequent injection of drug-free silicone elastomer mix into the molds produces rings having overmolded cores partially exposed to the external environment *via* orifices in the sheath. The shape and size of the orifices depends on the mold design. D – Four different types of exposed-core vaginal rings were produced: ring D1 contains a single small orifice; ring D2 contains six small orifices, three on each side of the ring; ring D3 contains 24 small orifices, twelve on each side; ring D4 contains two large window orifices, one on each side. E – representation of the ring cross-section.

Silicone elastomer rings, comprising cores loaded with HPMC and either lysozyme (a model protein) or lyophilised 5P12-RANTES powder and with the cores partially exposed to the external environment by an overmolded silicone elastomer sheath containing one or more discrete windows, were manufactured using a simple two-step injection molding process. ¼- or full-length cores (cross-sectional diameter, 4.2 mm) were first manufactured using a temperature-controlled laboratory-scale injection molding machine fitted with a custom mold assembly (Fig. 2).

DDU-4320 addition-cured silicone elastomer kit is comprised of two parts. Both parts contain a basic silicone formulation, primarily vinyl-functionalized and hydroxy-terminated poly(dimethyl siloxane)s. Part A also contains a platinum catalyst and part B contains a hydride-functionalized poly(dimethyl siloxane) crosslinker which when mixed react *via* a hydrosilylation addition reaction. Here, Part A and Part B DDU-4320 silicone elastomer premixes containing 8.58% *w*/w lysozyme and 17.2% w/w of 10 kDa or 120 kDa HPMC (bulk, >125 μm, 90–125 μm, 53–90 μm or < 53 μm particle size) were prepared by adding weighed quantities of the powdered materials into a screw-cap polypropylene container, followed by addition of the silicone elastomer parts, and then mixed using a Dual Asymmetric Centrifuge (DAC) mixer (30 s, 3000 rpm; SpeedMixer™ DAC 150 FVZ—K, Hauschild, Germany) (Fig. 2A). A and B premixes were then combined in a 1:1 ratio, according to the following procedure: (i) equal weights of each premix were added to a screw-cap polypropylene container to a final batch weight, (ii) the material was hand-mixed for 30 s and then DAC mixed (15 s at 1500 rpm). The active mix was transferred to a 75 g polypropylene SEMCO injection cartridge designed for use with the SEMCO Model-850 injection system. Full-length ring cores (outer diameter, 54.0 mm) were prepared by injecting the active mix into the heated ring mold assembly (85 °C) and curing for 3 min (Fig. 2B). The resulting lysozyme-loaded cores were subsequently overmolded with drug-free DDU-4320 silicone elastomer in a further injection molding step, using a custom mold assembly designed to introduce 24 circular orifices in the sheath (Fig. 2C and D3). Rings were subsequently demolded, deflashed (where necessary) and stored at ambient temperature until further testing.

¼-length DDU-4320 silicone elastomer cores containing 8.58% *w*/w freeze-dried 5P12-RANTES and 17.2% w/w 120 kDa HPMC (not sieved) were similarly manufactured and overmolded. Using custom mold assemblies, three alternative sheath designs were prepared to expose different fractions of the underlying ¼-length core. The final rings contained a ¼-length core having one or six discrete 3.0 mm diameter circular orifices (Fig. 2 D1 and D2; 9.34 or 56.04 mm^2^ core exposure), one ¼ core with two large windows (Fig. 2 D4; 210.9 mm^2^), or two ¼ -length cores with four large windows (see graphical abstract; 421.8 mm^2^).

#### 2.1.4 Rheological assessment of silicone elastomer mixes

Continuous flow rheology was carried out using an AR 2000 Rheometer fitted with a 40 mm diameter steel parallel plate (TA Instruments, USA) to assess the cure characteristics of the silicone elastomers mixes, following methods described previously [43,44]. Briefly, samples were applied to the base plate and the steel parallel plate was lowered to a gap depth of 1000 μm. Excess material was removed before the test. Following a 5 min equilibration step flow rheology was carried out at 21 °C, continuous ramp mode, from 0.01–100 Hz. Storage modulus (G'), loss modulus (G") and tan δ values were measured as a function of time.

#### 2.1.5 In vitro release testing of vaginal rings

Rings were placed in 15 mL of Type 1 water (Millipore Direct-Q 3 UV Ultrapure Water System, Watford, UK) in polypropylene containers (Synergy Devices Limited, United Kingdom). The containers were stored in an orbital shaking incubator (37 °C, 60 rpm, 25 mm orbital throw) and the release media were sampled periodically with complete volume replacement. Samples were stored at 4 °C and the concentrations of 5P12-RANTES measured using HPLC and/or ELISA.

#### 2.1.6 Quantification of 5P12-RANTES using HPLC

5P12-RANTES and lysozyme concentrations in the *in vitro* release samples were measured using a Waters Alliance e2695 HPLC and UV 2489 detector (Waters, Dublin, Ireland), Empower data handling software and an ACE C8 column (ACE 3 μm, C8, 300 Å, 150 × 4.6 mm) (Aberdeen, UK). 5P12-RANTES analysis was performed using 100 μL injection volumes, a gradient method comprising mobile phases A (0.1% TFA in water), B (0.0955% TFA/30% acetonitrile in water) and C (0.085% TFA/95% acetonitrile in water), and a column temperature of 37 °C. Lysozyme analysis was performed using 10 μL injection volumes, a gradient method comprising mobile phases A (0.1% TFA in water), B (0.1% TFA in acetonitrile) and a column temperature of 45 °C. For both methods, mobile phase flow rate was 1 mL/min and detection wavelength was 280 nm.

#### 2.1.7 Quantification of 5P12-RANTES using ELISA

5P12-RANTES concentrations were also quantified by ELISA using the Human CCL5/RANTES ELISA kit (R&D Systems, UK, cat no. DRN00B). Samples were analyzed as per the manufacturer's instructions and the appropriate dilutions were made to ensure all readings fell within range of the standard curve of the kit (0.002–2 ng/mL). The absorbance was measured at 450 nm (EnSpire™ Multimode Plate Reader, PerkinElmer, USA) with correction at 570 nm to account for plate imperfections. A four-parameter logistic (4-LP) standard curve was generated and sample concentrations were calculated relative to it. Results were subsequently multiplied by the appropriate dilution factor.

#### 2.1.8 Determination of release medium uptake

Ring weights were recorded before and after *in vitro* release testing. The rate of swelling of the rings was reported as a percentage increase in weight relative to the initial weights.

#### 2.1.9 SDS-PAGE

SDS-PAGE was performed using a Mini Protean II Tetra cell (Bio-Rad Laboratories, USA) using a 1× tris/glycine/SDS buffer. Samples were mixed at a 3:1 ratio with 2× Laemmli sample buffer and then wet-loaded onto a 20% Mini-PROTEAN TGX precast gel alongside a pre-stained molecular weight marker. Samples were run for 45 min, 150 V, and visualized using Coomassie staining.

#### 2.1.10 Physical characterization and mechanical testing of rings

Batches of rings were manufactured and their mechanical properties evaluated using a range of custom mechanical tests [45]. The force required to compress ring devices by 5 mm was measured using a Texture Analyser (TA-XTPlus, Stable Microsystems, UK), fitted with a 30 kg load cell and Texture Exponent (TEE32) 32 software, and using a custom ring holder. Full ring devices were tested in three different orientations due to the asymmetric nature of their designs, with core inserts being either at the top, side or base during each test. A number of other tests were performed on the rings, loosely derived from ISO 8009. A static, 28-day compression test was performed by placing rings into a custom compression jig that compressed them to 25 ± 5% of their original outer diameter (OD). After 28 days, the rings were removed from the compression jig and allowed to recover for a period of 15–20 s before the percentage recovery of the original ring diameter was recorded. Rings were subjected to a 1000-cycle compression test by compressing to 25 ± 5% of their original OD and releasing 1000 times. Custom ring holders were fabricated, capable of holding multiple rings simultaneously, for use with the Texture Analyser. An aluminium plate with multiple rectangular ring holding grooves (7 per plate) was mounted on the lower fixed platform of the analyser. An upper Perspex plate with identical grooves, mounted on the upper moveable arm of the TA (so the centrelines of lower and upper grooves were aligned), was lowered until it touched the top of the rings so that all rings were held in a vertical position with slight pre-compression. Generally, rings (*n* = 6 per formulation) were cyclically compressed from 100% to 25 ± 5% of their original OD, 1000 times at a crosshead speed of 15 mm/s. Finally, the degree of twist during compression was recorded. A custom manufactured twist-tester kept the top of the ring rotationally fixed while the bottom of the ring was able to rotate freely in a holder mounted on a low friction bearing. Rings were compressed by lowering the TA cross-arm so that the distance between the lower ring mount and the cross-arm was in accordance with diaphragm twist-test parameters as stated in Annex F of ISO8009:2014. Where ring external diameters did not correspond with those stated for diaphragm devices, the distance between the cross-arm and the ring holder was determined by extrapolation or interpolation using the mean external diameter for each ring formulation. The degree of twist (angular rotation) of the ring during compression was indicated by movement of a pointer, attached to the bottom holder, on an angular scale away from the starting (zero) position in either a clockwise or anticlockwise direction.

#### 2.1.11 Pharmacokinetic evaluation of 5P12-RANTES vaginal ring in sheep

Two exposed-core vaginal ring formulations – one containing a single 63.5 mg 5P12-RANTES core and two windows, the other containing two 63.5 mg 5P12-RANTES cores and having four windows – were evaluated for pharmacokinetics in Suffolk Cross sheep (36–37 months; 63.0–95.0 kg) at Envigo (Huntingdon, UK). Vaginal fluid, vaginal tissue and serum concentrations of 5P12-RANTES were measured during 28-day ring use. The sheep were housed in indoor pens with wheat straw bedding, and provided with natural light supplemented with overhead fluorescent lighting as necessary and full fresh air. The animals grazed on ewe and lamb pencil pelleted diet, were provided good-quality meadow hay *ad libitum*, and had an unrestricted water supply. A total of eight sheep were used in the study. On day 1 of the study, each animal was restrained in a comfortable standing position and the test ring inserted carefully into the cranial vagina, using a gloved finger and a small amount of medical-grade lubricant gel as appropriate. Strings (medical-grade monofilament suture material) were fitted to the rings before application and the ends of the tapes left hanging outside the vagina after application. These strings served as an external visual check that the ring was still in place and had not been expelled, and aided retrieval of rings at the end of the study period.

Blood and vaginal fluid were sampled at the following timepoints: pre-dose, 4 and 24 h, 2, 4, 7, 14, 21, 28 and 35 days post ring insertion. Blood samples were drawn from the jugular vein and incubated for a minimum of 60 min at room temperature to facilitate clotting. Subsequently, the samples were centrifuged (1500 × g, 15 min) to separate the serum and then divided and stored at −20 °C in polypropylene tubes. Vaginal fluid was sampled using a preweighed Weck-Cel sponge that was again weighed after fluid uptake. The sponge was held in place in the vagina for 1 min and stored at −20 °C in borosilicate glass tubes. Vaginal tissue biopsy specimens (5 mm diameter) were sampled on day 7, 21 and 35 post ring insertion from the dorsal aspect of the vagina under local anaesthetic (isoflurane and oxygen were used where necessary). Analgesia was administered 30 min prior to vaginal biopsy specimen collection. Samples were rinsed with RPMI 1640 and weighed prior to freezing at −70 °C. Frozen samples were submitted for analysis to Envigo's Department of Biomarkers, Bioanalysis, and Clinical Sciences (Immunoassay). Briefly, all tissue samples were homogenized in homogenization buffer (PBS with 2% Triton X-100 and protease inhibitor cocktail) at 4 °C and kept on wet ice at all times. The tissue specimens were placed in a minimum of 3 mL of buffer, and the ratio of buffer/sample weight was recorded. Samples were homogenated (GentleMACS dissociator) and centrifuged at 4566 × g for 10 min. The supernatant was collected into tubes (Watson labelled PP) and placed on wet ice or stored at −70 °C. Analysis was performed using a human CCL5/RANTES ELISA kit, according to the manufacturer's instructions. Clinical condition, body weight, and food consumption were recorded during the study.

## 3 Results and discussion

Polymeric systems for the sustained release of large molecular weight therapeutic agents – and particularly proteins – has been a major topic of interest in the pharmaceutical sciences field since the 1970s [[46], [47], [48], [49], [50], [51], [52]]. More recently, delivery systems for sustained release of proteins have been reported using hydrogels [53,54], injectable biodegradable systems [55,56] and electrospun biodegradable fibers [57]. Implantable silicone elastomer systems for the sustained/controlled release of proteins have also previously been reported, and mostly involve incorporation of high concentrations of one or more water-soluble or pore-forming (porosigens) excipients into the silicone matrix [[58], [59], [60], [61], [62], [63], [64]]. However, a particular difficulty with this approach is that the rings imbibe fluid and swell in proportion to the quantity of excipient incorporated. Since relatively large quantities of excipient are required to obtain meaningful release of proteins, the ring devices can often increase significantly in size and weight [58,59]. Clearly, this is impractical and unsafe from a clinical perspective. To overcome this difficulty, vaginal ring devices have been reported in which one or more small drug-loaded module(s) containing the drug and various water-soluble or water-swellable excipients are manufactured separately and later inserted into a silicone elastomer ring body containing channels that act as retainers for the modules [[26], [27], [28]]. In a similar fashion, a ‘flux controlled pump’ capable of 30-day controlled release of the model macromolecules dextran and insulin to the vaginal mucosa has previously been reported [65,66]. The pump comprised a compressed water-soluble polymer pellet compounded with a macromolecular drug and enclosed in a hard polymer casing containing orifices to allow influx of vaginal fluid and efflux of the hydrated contents. The hydration kinetics and osmotic pressure were controlled through orifice number and geometry. In order to retain the pump in the vagina without resorting to surgical stitching, the pump was attached to a separate ring-shaped polyurethane retainer. In this way, the ring retainer is physically distinct from the drug delivery module. However, both these approaches have their disadvantages. In the former, there is limited loading capacity in the relatively small modules for the drug and excipient (although multiple inserts are possible) and the duration of release is also constrained. In the latter, the design is inelegant and impractical for use in women. The ring design described in this current study successfully builds on the principles and concepts described in these previous studies while addressing the manufacturing and design obstacles.

#### 3.1.1 Rheological assessment of the active silicone mixes

**Fig. 3:**
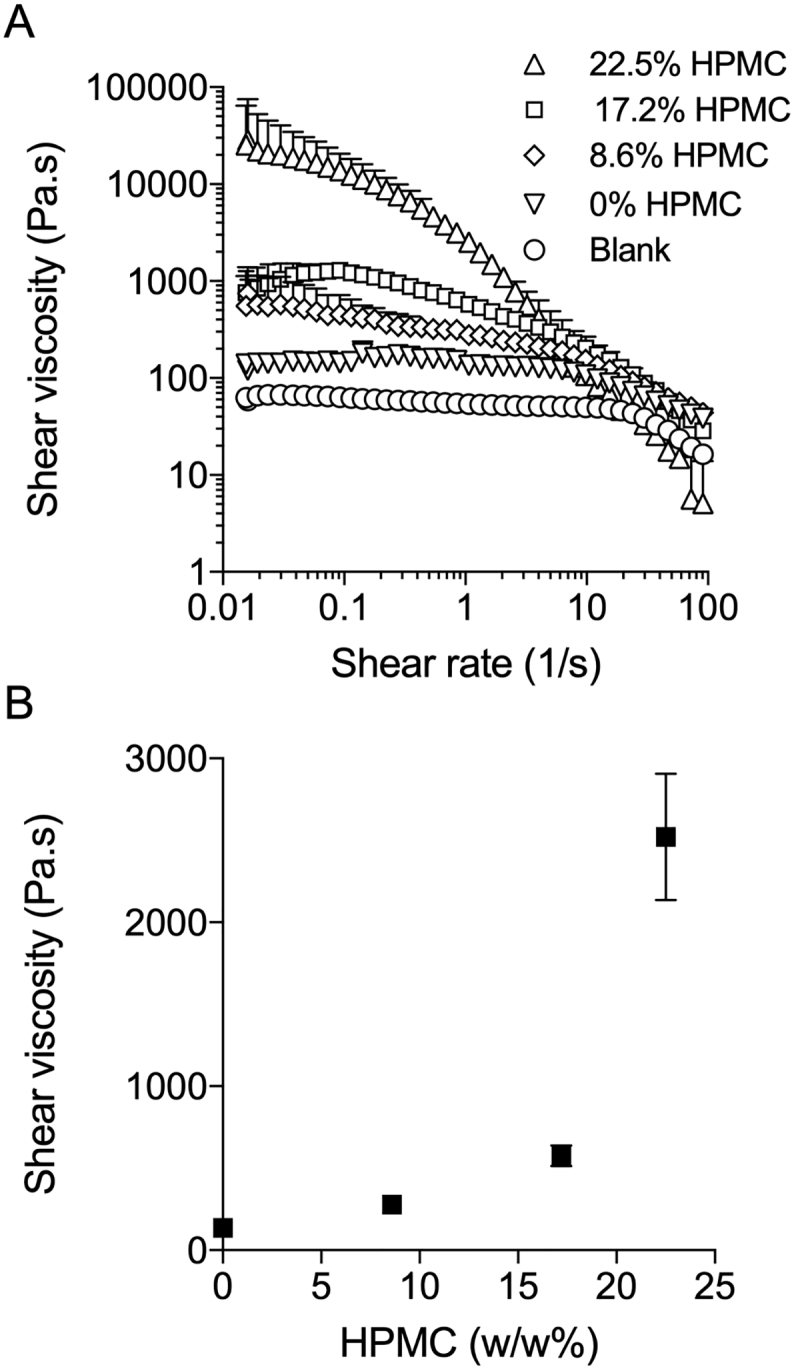
Mean shear viscosity (± SD; *n* = 3) of DDU4320 liquid silicone rubber homogenous mastermix formula-tions at a shear rate range of 0.01–100 Hz (A) and at a shear rate of 1.065 Hz (B). Formulations contained 0%, 8.6%, 17.2%, and 22.5% *w*/w HPMC loading and 8.6% w/w lysozyme. The blank formulation consisted of 100% w/w DDU4320.

The protein-loaded cores of the rings were manufactured by injection molding of liquid silicone elastomer mixes containing either lysosyme or lyophilised 5P12-RANTES and HPMC. The viscosities of these mixes depend upon the viscosity of the silicone elastomer used and the concentration and particle size of the incorporated drugs and excipients. The liquid silicone rubber – immediately following mixing of all components and up to the point of molding – can be considered as an inelastic non-Newtonian liquid, the viscosity of which is important for injection into the molds. Rheological measurement of the shear viscosity as a function of increasing shear rate is reported in Fig. 3 for blank DDU-4320 and four formulations containing loadings of HPMC ranging from 0 to 22.5% *w*/w. As the loading of HPMC is increased in the formulations, there is a reduction in the shear rate required to observe shear thinning in the liquid silicone continuous phase, indicating the particles of HPMC are not interacting with the silicone. Additionally, the effect of HPMC loading on viscosity is significantly higher at lower shear rates where the flow is Newtonian compared to shear rates of >10, where the curves begin to become parallel, follow a power-law behaviour and show similar viscosities across the formulations. The manual process used to inject the silicone into the mold cavity produces a low shear rate at which the mixture is observed to be in Newtonian flow and the particle loading is critical to viscosity. In this study, where rings were manufactured *via* a manual injection molding process, the maximum HPMC concentration that could practically be injected was 17.2% *w*/w. In larger scale LSR molding equipment having a hydraulic piston for injection of the silicone mix, typical shear rates are in the order of 20–700 Hz within the injection unit, and can increase up to 10,000 Hz within the mold tool depending on the geometry of the runners and gates used. These higher shear rates could facilitate the use of HPMC loadings greater than those possible using manual injection, due to the shear thinning nature observed for the formulations analyzed.

#### 3.1.2 Ring manufacture

Using custom mold assemblies containing protruding pins for location of the core component, vaginal rings with orifices in the silicone elastomer sheath that partially expose the underlying protein+excipient loaded silicone elastomer core were readily manufactured in two steps *via* conventional injection molding technology (Fig. 2). Rings were manufactured having 1, 6 or 24 small circular orifices or two large windows, each giving rise to different surface areas of exposure of the underlying core (Table 2). Further modulation of the surface area of exposure could be readily achieved by design and fabrication of mold assemblies with different pin configurations.

**Table 2:** Characteristics of vaginal ring formulations containing either the model protein lysozyme or the experimental CCR5 inhibitor 5P12-RANTES.

Vaginal ring formulation number	Drug (loading in mg)	Core length	Characteristics of HPMC in core			No. and size of orifices	Surface area of core exposure (mm^2^)
			HPMC MW (kDa)	HPMC loading (w/w%)	HPMC particle size (μm)		
1	–	full length (blank)	–	–	–	24 small	224.2
2	Lysozyme (234.23)	full length	120	8.6	bulk	24 small	224.2
3	Lysozyme (234.23)	full core	120	17.2	bulk	24 small	224.2
4	Lysozyme (234.23)	full core	10	17.2	bulk	24 small	224.2
5	Lysozyme (234.23)	full core	120	17.2	<53	24 small	224.2
6	Lysozyme (234.23)	full core	120	17.2	53–90	24 small	224.2
7	Lysozyme (234.23)	full core	120	17.2	90–125	24 small	224.2
8	Lysozyme (234.23)	full core	120	17.2	<125	24 small	224.2
9	5P12-RANTES (63.49)	¼ core	120	17.2	bulk	1 small	9.3
10	5P12-RANTES (63.49)	¼ core	120	17.2	bulk	6 small	56.0
11	5P12-RANTES (63.49)	¼ core	120	17.2	bulk	2 large	210.9
12	5P12-RANTES (126.98)	¼ core × 2	120	17.2	bulk	4 large	421.8

#### 3.1.3 In vitro release of model protein lysosome

**Fig. 4:**
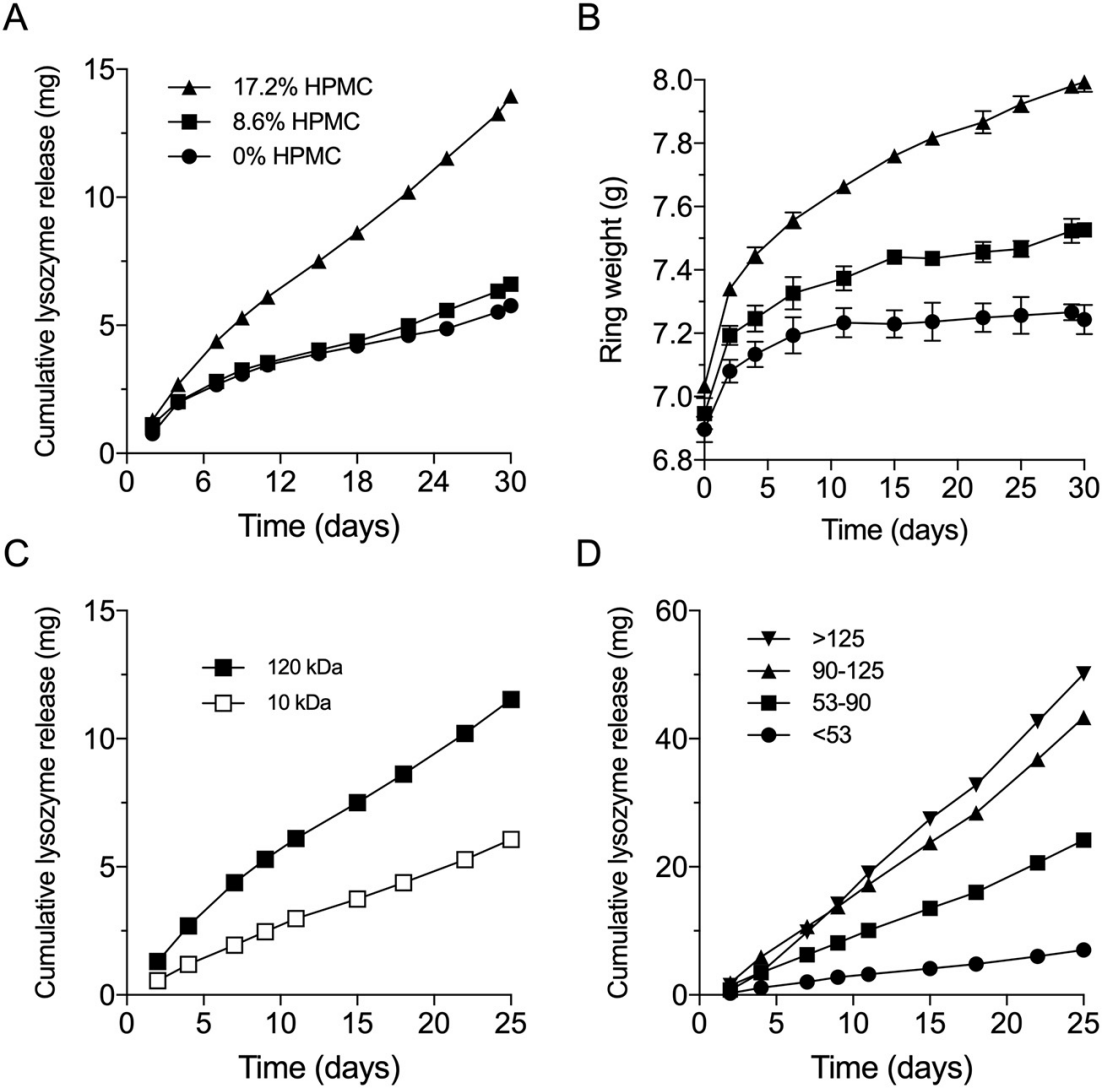
Effect of HPMC loading (120 kDa) on drug release (A) and ring swelling (B) on 24 orifice full core rings containing lysozyme. The Effect of HPMC molecular weight (C) and HPMC (120 kDa) particle size (D) on drug release from full core vaginal rings into water, 37 °C. Mean ± SD, *n* = 3.

Cumulative lysosome release *vs.* time plots for exposed-core vaginal rings (24 small circular orifices) containing 224.2 mg lysozyme in a full-length silicone elastomer core modified with either 0, 8.6 or 17.2% *w*/w bulk HPMC powder (ring formulations 1–3, Table 2) are presented in Fig. 4A. For the two lower HPMC concentrations, lysosome release rates were similar with a total of ~5 mg released over the 30-day period. A 3-fold increase in lysosome release (~15 mg after 30 days) was obtained by increasing the HPMC loading to 17.2% *w*/w. It is assumed that relatively high HPMC concentrations are needed to promote sufficient penetration of release medium into the silicone cores, resulting in the formation of interconnected aqueous pores and channels in the silicone elastomer and a concomitant increase in the drug release rate, as has been reported previously for other excipient-modified silicone elastomer drug delivery systems [26,58,63]. The ring weights increase over the course of the *in vitro* release study (Fig. 4B) due to imbibing of the release medium into the ring, are dependent upon the HPMC content, and correlate with the *in vitro* release data (Fig. 4A). The molecular weight (Fig. 4C) and the particle size (Fig. 4D) of the HPMC excipient incorporated into the silicone elastomer core of the vaginal ring also significantly influenced the release of lysozyme. A 120 kDa HPMC material increased release twofold compared with a 10 kDa HPMC (Fig. 4C), while increasing HPMC particle size from <53 μm to >125 μm produced a 7-fold increase in lysosome release, reflecting similar trends reported previously for release of the model protein drug interferon from simple silicone elastomer matrices [58].

#### 3.1.4 *In vitro* release of 5P12-RANTES

**Fig. 5:**
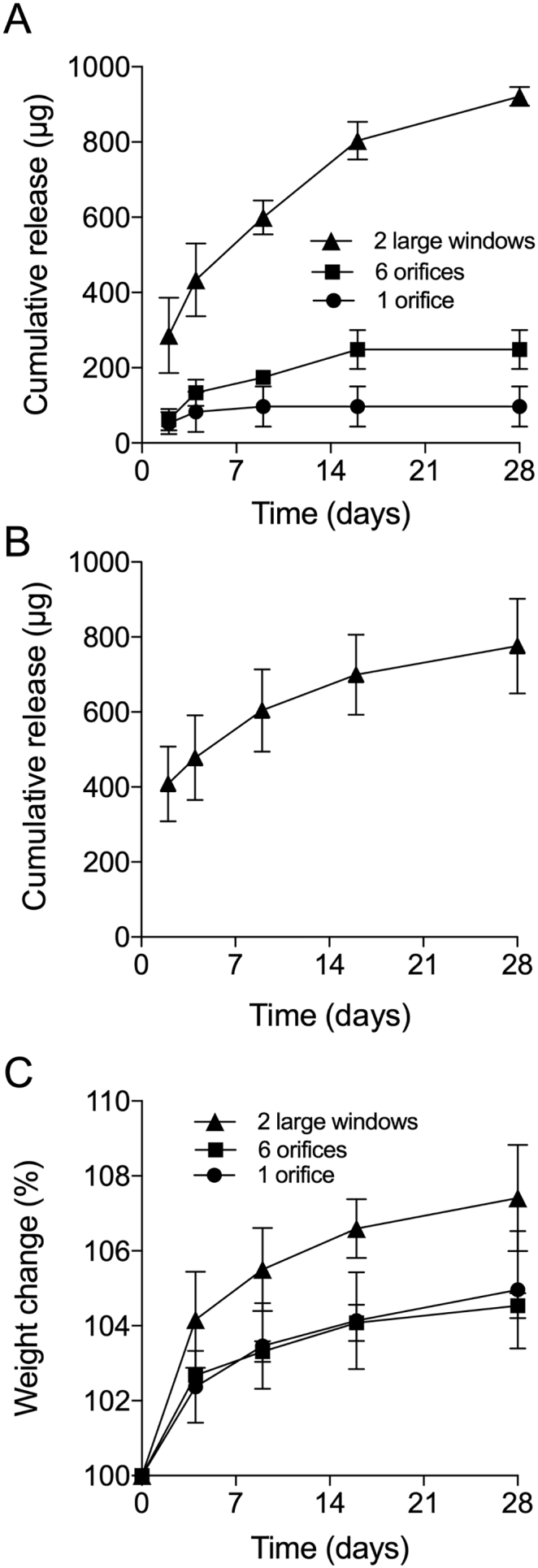
Cumulative release of 5P12-RANTES from two large window, six orifice and one orifice exposed-core vaginal rings as measured by HPLC (A) and cumulative release of 5P12-RANTES from two large window exposed-core vaginal ring as measured by ELISA (B). Swelling of the rings was measured by recording as percentage weight change (C). Samples were incubated in water during the study, 37 °C, mean ± SD, *n* = 3.

Unlike the lysosome release study, limited availability of 5P12-RANTES constrained *in vitro* release testing to rings with only ¼-length cores. HPLC-generated data for *in vitro* cumulative release of 5P12-RANTES from ¼-core rings having 1 orifice, 6 orifices or 2 large windows (Formulations 9–11, respectively; Table 2) are presented in Fig. 5A, and demonstrate that increasing the surface area of core exposure to the release medium leads to increased 5P12-RANTES release. For the two-window ring, almost 300 μg 5P12-RANTES was released on Day 1, with 921 μg (equivalent to 1.45% of the initial 63.5 mg loading) released in total over the 28-day test period. For comparison, the lead candidate 25 mg dapivirine (DPV) ring – a matrix-type ring which successfully completed Phase III clinical testing in 2016 [8,9] and is currently undergoing regulatory review – provides Day 1 *in vitro* DPV release of ~ 600 μg and total cumulative release of ~4500 μg (18% of the initial 25 mg loading), reflecting the much greater surface area for drug release offered by the traditional matrix-type ring format and the greater permeability of DPV in the silicone elastomer system due to its physicochemical properties (Fig. 1). In comparison to VRC01 drug release from pod-type IVRs, 5P12-RANTES release is much lower. This is likely related to the differences in ring formulation. 5P12-RANTES drug release is dependent on water imbibing and swelling the HPMC component which subsequently facilitates the dissolution and release of 5P12-RANTES from the silicone elastomer core. It is likely that drug release is constrained by the viscosity and polymeric nature of the core. Conversely, as polymeric excipients are not used in pod-ring design the drug, *e.g.* VRC01, is in a more readily available state. Two methods to improve the drug release would be to increase the HPMC loading percentage of the core or use a half- or full- core ring. Indeed, in the PK study here we included a half-core arm with the aim to further enhance drug release. Fig. 5B shows *in vitro* 5P12-RANTES release data for the 2-window ring (Formulation 11, Table 2) as measured by ELISA. The values are broadly similar to those measured by HPLC (Fig. 5A), and confirm that that 5P12-RANTES effectively maintains its stability during ring manufacture such that antibody binding, and thus conformational integrity, is retained. Further evidence that 5P12-RANTES is released intact from the vaginal rings and that it remains stable in the ring upon storage at 4 °C for 3 months is provided by the SDS-page data presented in Fig. S1 (Supplementary Information). *In vitro* 5P12-RANTES release data, measured both by HPLC and ELISA, after 3-month storage is presented in Fig. S2 (Supplementary Information), again demonstrating good stability of the protein and reproducible release rates. It is anticipated that there would be no detrimental effects to the rings at room temperature, and potentially higher environmental temperatures, over time as it was previously reported that 5P12-RANTES is stable following incubation at 55 °C for 24 h and 40 °C for 7 days [37]. However, this requires further investigation.

#### 3.1.5 Mechanical testing

**Fig. 6:**
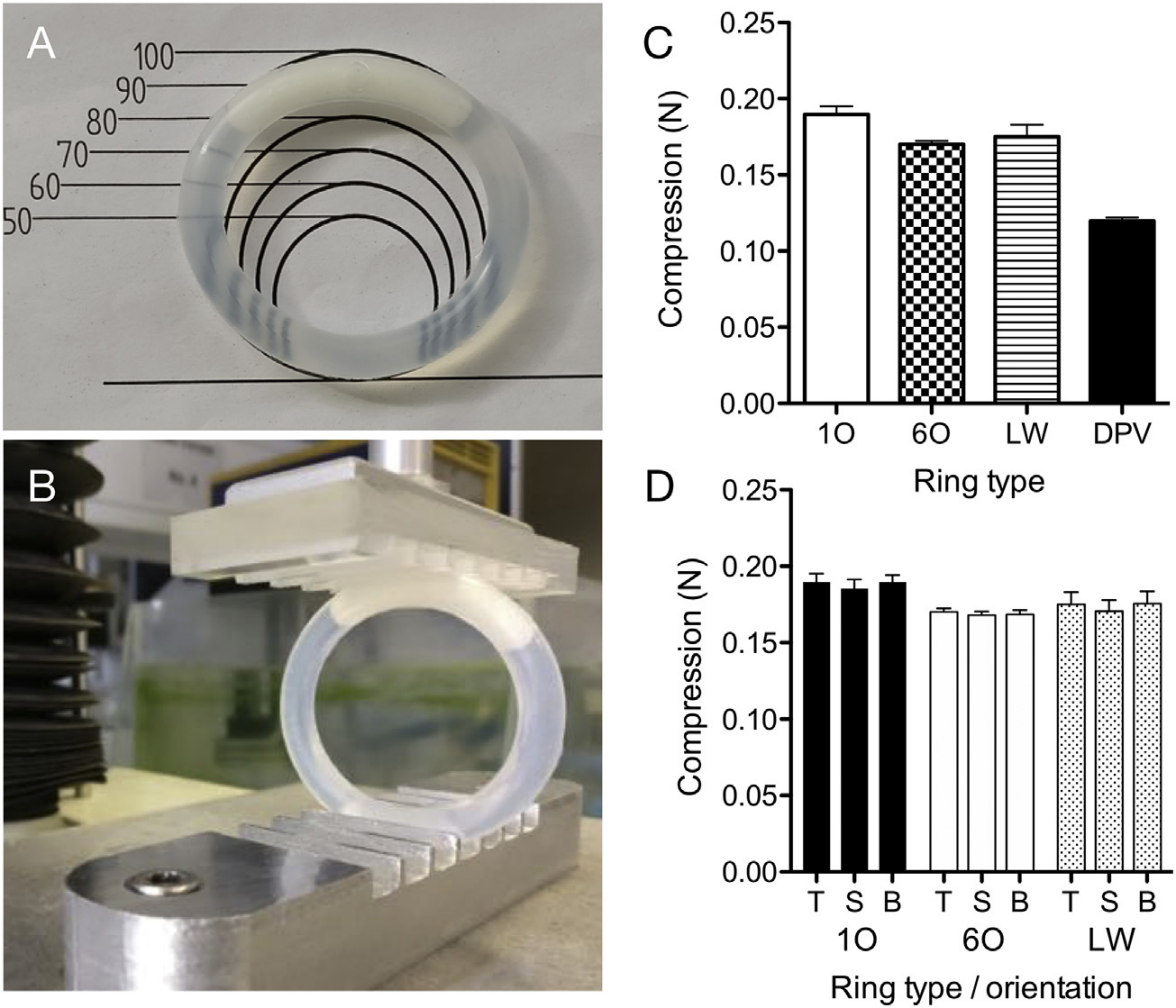
Ring following 28-day static deformation testing, the one orifice ring pictured recovered 90–100% of its outer diameter upon removal from the jig (A); ring prior to commencing 5 mm compression test (B); 5 mm compression testing of one orifice (1O), six orifice (6O), two large windows (LW) and a comparator 25 mg dapivirine ring manufactured in-house (C); 5 mm compression testing of rings in various orientations: T – top, with the core segment positioned upward toward the probe (as pictured in b); S – side, with core segment facing out from the instrument; B – bottom, with the core positioned downwards furthest from the probe (D).

Fig. 6C reports mean forces to produce 5 mm compression in ¼-core ring devices. A decrease in force was observed from 0.19 to 0.17 N as the number of orifices increased from one to six. For the dual large window ring, the force required to compress by 5 mm was between that for one or six orifice designs at 0.18 N. These values were significantly lower than those observed for commercial rings, Fertiring® and Progering® at 0.89 and 1.24 N respectively indicating that the sheath material in the RANTES ring is of a lower modulus. The orientation of the ring during 5 mm compression did not produce statistically significant differences in the mean force value (Fig. 6D), suggesting that the clinical positioning of the ring device would not be critical from a user comfort perspective.

Similar results were observed for rings undergoing either static, 28-day compression or dynamic, 1000-cycle compression testing. In both cases, rings recovered to at least 90% of their original outer diameter following removal from the corresponding jig and there was no evidence of damage at the core/membrane orifice interface, or at any other points throughout the devices. There was no observable correlation between the ring orifice geometries and the degree of mean angular deviation recorded during twist tests. Critically, there were no failure modes observed in any of the devices during any of the mechanical tests.

#### 3.1.6 Sheep pharmacokinetics

**Fig. 7:**
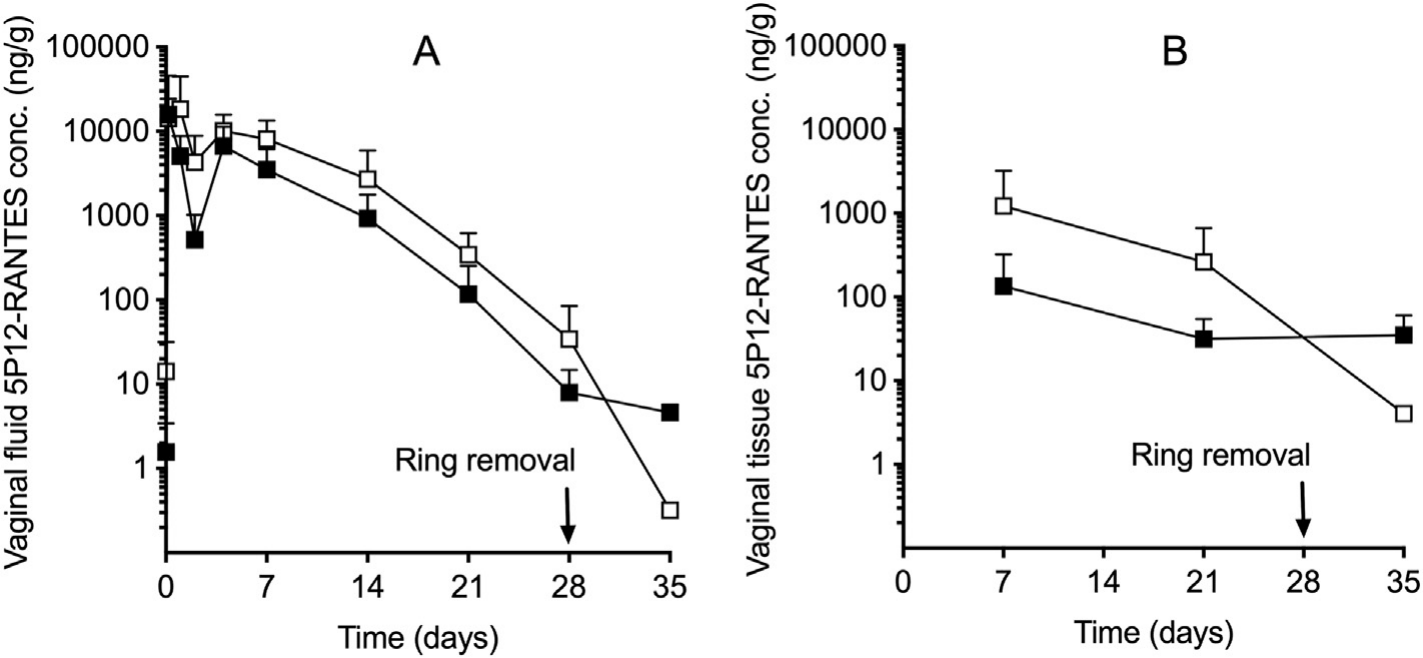
Concentrations of 5P12-RANTES measured in the vaginal fluid (A) and biopsied vaginal tissue (B) of Suffolk Cross sheep during and after 28-day vaginal ring use. Black plot symbols show data for ¼-core rings with two large windows; unfilled plot symbols show half core rings with four large windows. Plot symbols represent mean ± standard deviation of four replicates.

No clinical signs were observed throughout the study and all rings remained in place for the 28-day treatment period. The serum concentrations of 5P12-RANTES were consistently below the limit of quantification (<70.4 pg/mL), unlike those measured previously for 5P12-RANTES vaginal gels [41]. 5P12-RANTES was detected in vaginal fluid and vaginal tissue at all sampling timepoints post-dosing for both the single ¼-core ring and the double ¼-core ring (Formulations 11 and 12, respectively; Fig. 7). For these rings, vaginal fluid concentrations of 5P12-RANTES peaked (*C*_*max*_) within 24 h at 19.6 and 27.1 μg/g, respectively, followed by steadily declining concentrations thereafter. By Day 28 (day of ring removal), 5P12-RANTES concentrations had declined to 8.0 and 34.3 ng/g, respectively (Fig. 7). One week after ring removal (Day 35), 5P12-RANTES was still detectable in vaginal fluid, albeit at significantly decreased levels (0.32 and 4.6 ng/g, respectively). Pharmacokinetic statistical parameters *C*_*max*_, *T*_*max*_ and *AUC*_*0-last*_ are presented in Table 3 for the vaginal fluid data. The rate (*C*_*max*_) of local exposure of female sheep to 5P12-RANTES increased with increasing dose over the dose range 63.49–126.98 mg. However, the increases were slightly less than the proportionate dose increment. At the highest dose level (126.98 mg), the *C*_*max*_ values were 31% lower than those values predicted from a linear relationship. The extent (*AUC*_*0-last*_) of local exposure of female sheep to 5P12-RANTES increased approximately proportionately (AUC ratio 1:2.1) with increasing dose over the dose range 63.5–127 mg. Vaginal tissue concentrations of 5P12-RANTES during the period of ring use ranged between 32 and 135 and 260–1220 ng/g for vaginal rings containing low (63.49 mg) and high (126.98 mg) initial loadings of 5P12-RANTES, respectively (Fig. 7B). Overall, the 5P12-RANTES concentrations measured in vaginal fluid and tissue are at least 50 and up to 50,000 times the reported 18 pM (0.142 ng/g) *in vitro* IC_50_ value [42].

**Table 3:** Statistical pharmacokinetic parameters for 5P12-RANTES measured in sheep vaginal fluid and vaginal tissue during and after 28-day use of exposed-core vaginal rings. *C*_max_ and *AUC*_*0 –last*_ values are presented as mean ± SD of four replicates. *T*_max_ values are presented as the medians and ranges of four replicates.

	Vaginal fluid			Vaginal tissue		
5P12-RANTES vaginal ring formulation number (see Table 2)	*C*_max_ (μg/g)	*T*_max_ (days)	AUC_0-last_ (*μ*g.day/g)	*C*_max_ (ng/g)	*T*_max_ (days)	AUC_0-last_ (*μ*g.day/g)
11	19.6 ± 27.5	1, 0.17–4	53.3 ± 37	149 ± 176	14, 7–35	1.5 ± 1.4
12	27.1 ± 22.4	0.58, 0.17–7	117.0 ± 54	1233 ± 1980	7, 7–21	11.9 ± 2.0

Unsurprisingly, pharmacokinetic concentrations of 5P12-RANTES obtained with these sustained release vaginal rings were significantly lower than those reported previously for aqueous vaginal gel formulations in the sheep model, where vaginal fluid concentrations declined from a high of 10^6^ to 10^7^ ng/g at 1 h and 10^2^ to 10^4^ ng/g at 96 h post dosing and vaginal tissue concentrations ranged from 10^5^ to 10^6^ ng/g at 12 h post dose [41].

## 4 Conclusions

This new, simple to manufacture, vaginal ring design – comprising one or more drug-loaded cores exposed to the external environment *via* orifices or windows in the overmolded sheath – has demonstrated potential for sustained release of the model protein lysosome and the antiretroviral protein 5P12-RANTES, and presumably might also be useful for release of other macromolecular drugs. The incorporation of relatively high concentrations of the hydrophilic pharmaceutical excipient HPMC into the silicone elastomer cores enhances the influx of release medium, thereby significantly enhancing the release rate of the incorporated protein.

The ring design also lends itself to practical incorporation of additional drug substances, most notably other antiretrovirals to produce a combination microbicide product and/or a contraceptive steroid to produce a long-acting MPT product. For example, in addition to inclusion of 5P12-RANTES in the core, incorporation of DPV and/or levonorgestrel (LNG) into the sheath component would likely provide similar *in vitro* and *in vivo* release characteristics as the current 25 mg DPV Ring-004 or the DPV + LNG currently being evaluated in the clinic [8,9,67,68]. In this way, novel combination antiretroviral vaginal rings could be considered, incorporating and releasing drugs having very different physicochemical properties.

## Author contributions

All authors contributed to the design of experiments and analysis of the data. J.W.M. and staff at Envigo conducted most of the experimental work. P·B. designed and fabricated the vaginal ring injection molds. V.L.K. was responsible for lyophilization of the 5P12-RANTES. O.H. and R.E.O. helped develop the HPLC and ELISA methods for quantification of 5P12-RANTES. The manuscript was drafted primarily by R.K.M., J.W.M, and P.B, with input and review from other authors.

## Conflict of interest

The authors declare no competing financial interest.

## Appendix A Supplementary data

Supplementary material Image 1
