# Supplementary material for: Vaginal rings with exposed cores for sustained delivery of the HIV CCR5 inhibitor 5P12-RANTES
Source: J Control Release. 2019 Mar 28;298:1–11. doi: 10.1016/j.jconrel.2019.02.003 (PMC6414755; doi:10.1016/j.jconrel.2019.02.003)
Supplement: Supplementary file 1 — Supplementary material [file mmc1.docx]

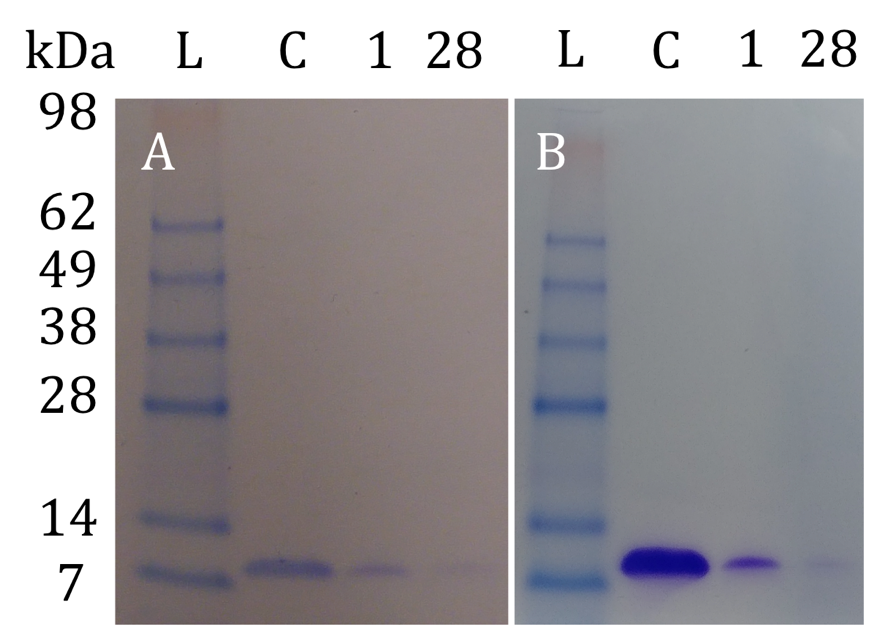


**Fig. S1.** SDS-PAGE of 5P12-RANTES released into water, 37°C, during in vitro release testing from exposed-core vaginal rings with large orifice windows (A) and again following 3 months stability storage at 4°C (B). Results represent first and last sampling time-point, n=3. L: Protein ladder (SeeBlue® Plus2 Prestained Standard); C: 5P12-RANTES positive control; 1: day 1 sample; 28: day 28 sample.

 
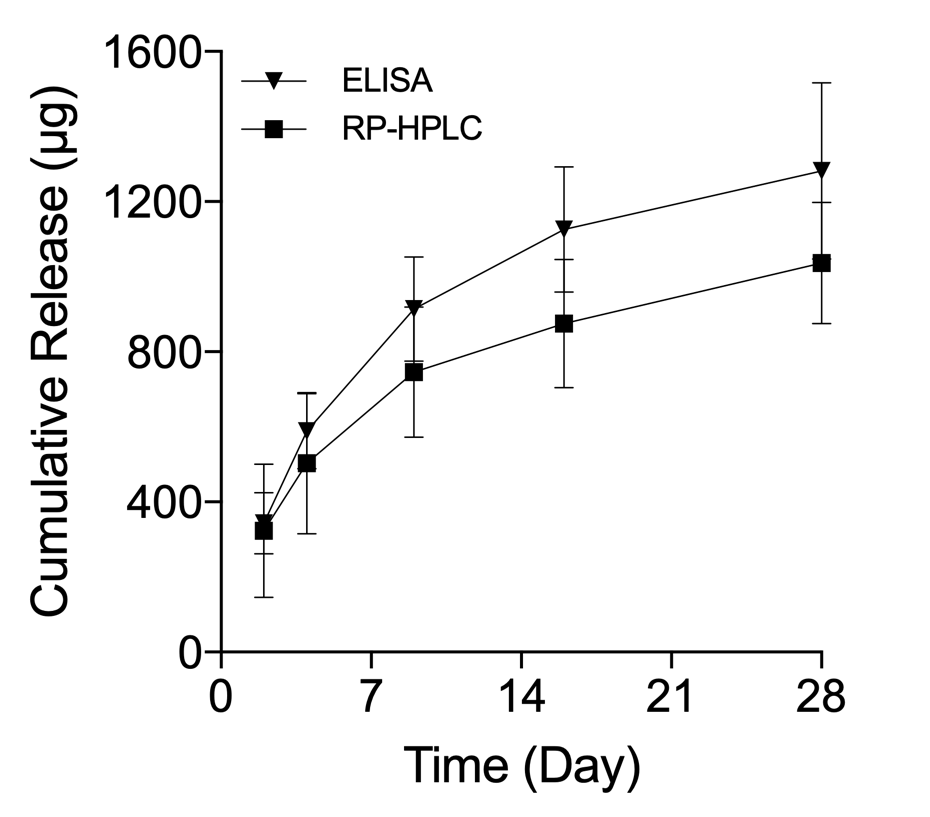


**Fig. S2.** Cumulative release of 5P12-RANTES from exposed-core vaginal rings with a large window orifice following 3-month stability storage at 4°C as measured by HPLC and ELISA. In vitro release testing was performed using water, 37°C, Mean ± SD, n = 3.

**Table S3.** Method for freeze-drying of 5P12-RANTES aqueous solution.

| **Thermal Treatment** | | | | |
| --- | --- | --- | --- | --- |
| Step No. | Temp (°C) | Time (min) | Vacuum (mTorr) | Condition |
| Step 1 | 5 | 10 | - | Hold |
| Step 2 | −40 | 60 | - | Ramp |
| Freeze Temp: | −40°C |  |  |  |
| Additional Freeze: | 120 min |  |  |  |
| Condenser Setpoint: | −50°C |  |  |  |
| Vacuum Setpoint: | 50 mTorr |  |  |  |
| **Primary Drying** | | | | |
| Step No. | Temp (°C) | Time (min) | Vacuum (mTorr) | Condition |
| Step 1 | −35 | 180 | 120 | Hold |
| Step 2 | −30 | 60 | 190 | Ramp |
| Step 3 | −30 | 180 | 190 | Hold |
| Step 4 | −25 | 60 | 190 | Ramp |
| Step 5 | −25 | 180 | 190 | Hold |
| Step 6 | 20 | 120 | 190 | Ramp |
| Step 7 | 20 | 360 | 190 | Hold |
| Post Heat | 20 | 600 | 50 | Hold |
